# The Role of Collagen in Coronary Artery Disease

**DOI:** 10.3390/ijms27219806

**Published:** 2026-10-31

**Authors:** Zihan Yang, Xianghuan Xie, Yuqing Shi, Yanghui Chen, Guangzhi Chen, Yang Sun, Renfan Xu

**Affiliations:** 1 Division of Cardiology, Department of Internal Medicine, Tongji Hospital, Tongji Medical College, Huazhong University of Science and Technology, 1095# Jiefang Ave., Wuhan 430030, China; mao12102023@163.com (Z.Y.); m202476596@hust.edu.cn (X.X.); yuqingshi2025@163.com (Y.S.); chenyh_c@tjh.tjmu.edu.cn (Y.C.); chengz@tjh.tjmu.edu.cn (G.C.); 2 Hubei Key Laboratory of Genetics and Molecular Mechanisms of Cardiological Disorders, Wuhan 430030, China; 3 Department of Medical Ultrasound, Tongji Hospital, Tongji Medical College, Huazhong University of Science and Technology, Wuhan 430030, China

**Keywords:** coronary artery disease, collagen, extracellular matrix remodeling, atherosclerosis, myocardial infarction, genetic variation

## Abstract

Coronary artery disease (CAD) remains a major cause of morbidity and mortality worldwide, imposing a substantial burden on patients and healthcare systems. Its development and progression are driven not only by lipid accumulation and inflammation but also by extracellular matrix remodeling. Collagen is a core component of the cardiovascular extracellular matrix. Its subtype composition, maturation, cross-linking, synthesis and degradation collectively shape the structural and functional properties of vascular and myocardial tissues. Here, we review collagen biology and use expression profiles from GTEx v11 and Heart Cell Atlas v2 to illustrate tissue- and cell type-specific patterns. These profiles illustrate the expression patterns and major cellular sources of the collagen gene family in the coronary artery and left ventricle. We also evaluate evidence linking collagen-related genetic variation to CAD susceptibility. By connecting genetic variation with protein processing and tissue architecture, this review delineates the potential roles of collagen in plaque stability, coronary thrombosis and post-infarction myocardial remodeling. This framework provides a basis for developing biomarkers of collagen remodeling and mechanism-guided therapeutic strategies.

## 1. Introduction

Cardiovascular diseases are a leading cause of disability-adjusted life years (DALYs) and mortality in the Global Burden of Disease (GBD) study. Among them, ischemic heart disease (IHD), which encompasses clinical conditions such as coronary artery disease (CAD), ranked as the leading cause of DALYs worldwide, accounting for approximately 193 million DALYs in 2023 [1]. Coronary atherosclerosis is the predominant substrate of coronary artery disease (CAD), although non-atherosclerotic etiologies also contribute [2]. The occurrence and progress of coronary atherosclerosis is accompanied by the continuous remodeling of the vascular wall extracellular matrix (ECM) [3]. After myocardial infarction (MI), the myocardial ECM undergoes further degradation, redeposition, and scar maturation, and its dysregulation can promote adverse ventricular remodeling and heart failure [4]. As a major component of the ECM, collagen is not only remodeled during disease progression but also actively shapes the remodeling process. The collagen family comprises 28 molecular types that assemble into fibrils, networks and other supramolecular structures with distinct biological functions [5].

In normal tissues, the distribution of collagen is closely related to the layered structure of coronary arteries and heart walls. The coronary artery supplies blood to the myocardium, and its wall is divided into intima, media, and adventitia from the inside out. Type IV collagen forms part of the basement membrane underlying endothelial cells and of the external lamina surrounding smooth-muscle cells. Types I and III collagen are distributed in the subendothelial connective tissue, smooth-muscle cells, and adventitia, forming fibrous scaffolds of the vascular wall [6,7]. The heart wall is composed of the endocardium, myocardial layer, and epicardium from the inside out in that order. In the normal mammalian myocardium, type I collagen predominates, accounting for approximately 85–90% of total myocardial collagen, whereas type III collagen contributes approximately 5–11% [4]. Within the myocardial layer, type I and type III collagen form a network of interconnected structures, forming the endomysial that surrounds and connects individual myocardial cells, as well as the perimysial matrix that surrounds myocardial cell bundles, providing structural support for the myocardium [4,8].

These ordered collagen structures undergo significant changes during the disease process. In coronary atherosclerotic plaques, collagen synthesis and accumulation help maintain the stability of fibrous caps, while an imbalance between synthesis and degradation may damage their integrity. For example, type VIII collagen can promote the migration of vascular smooth-muscle cells and facilitate the accumulation of type I collagen during the formation of fibrous caps [9]. After myocardial infarction, collagen remodeling also plays a crucial role. Due to the limited regenerative capacity of the adult myocardium, necrotic myocardial tissue is mainly replaced by scar tissue rich in collagen to maintain the structural integrity of the ventricular wall. Effective repair depends not only on the amount of collagen deposition, but also on its deposition time, spatial organization, and the mechanical strength of the formed matrix. Collagen maturation helps to enhance the stability of infarcted scars [10], and type V collagen derived from macrophages is involved in the assembly and arrangement of collagen fibers [11]. By contrast, persistent collagen deposition and lysyl oxidase-mediated cross-linking increase myocardial stiffness and can promote adverse ventricular remodeling [12].

In this review, we integrate data from publicly available databases with evidence from experimental studies to examine how collagen synthesis, processing and organization influence plaque stability, coronary thrombosis and post-MI repair [13,14]. This multidimensional framework aims to distinguish protective collagen responses from maladaptive remodeling and to identify new opportunities for the development of collagen-based biomarkers and therapeutic interventions.

## 2. Structure and Function of Collagens

Collagens are major components of the extracellular matrix (ECM), providing mechanical support to extracellular fibrillar networks while also mediating diverse biological functions through interactions with cell-surface receptors. Human genomic studies have identified 28 collagen types, each comprising a distinct combination of α-chains encoded by different collagen genes. On the basis of their molecular architecture, the continuity of their triple-helical domains and their modes of supramolecular assembly, collagens can be classified into several groups: fibrillar collagens, network-forming collagens, fibril-associated collagens with interrupted triple helices (FACITs), microfibrillar collagens, membrane-associated collagens with interrupted triple helices (MACITs), anchoring fibril collagens, beaded-filament-forming collagens, and multiple triple helix domains and interruptions (multiplexin). Through class-specific modes of molecular assembly, these collagens form intricate ECM networks that confer distinct mechanical properties and biological functions on different tissues. Notably, transmembrane collagens can also occur in soluble forms [5,15].

Overall, members of the collagen family are characterized by proline-rich Gly-X-Y repeating motifs and the triple-helical domain [16]. At the level of primary structure, collagens contain repetitive Gly-X-Y amino acid sequences, in which the X and Y positions are frequently occupied by proline and hydroxyproline. Interruptions within selected Gly-X-Y repeats confer local flexibility and conformational plasticity on collagen molecules [17]. On this basis, each α chain adopts a left-handed polyproline II-type helical conformation during biosynthesis beginning at the N terminus, and three α chains subsequently assemble from the C terminus into a triple-helical domain [18,19], forming the fundamental structural unit of procollagen and the most distinctive structural hallmark of collagens. Within the triple helix, glycine residues are positioned along the central axis, whereas interchain hydrogen bonds and stabilizing interactions associated with hydroxyproline residues enhance triple-helix stability [20,21]. At the quaternary structural level, following proteolytic maturation, collagen molecules assemble in a staggered manner into collagen fibrils, generating the characteristic D-periodicity of approximately 67 nm [22], and are further organized into collagen fibers through covalent cross-linking. This hierarchical organization provides the structural basis by which collagen maintains the integrity of connective tissues such as skin, bone, and blood vessels. Thus, through the hierarchical progression from Gly-X-Y repeats to single-chain helix formation, triple-helix folding, and fibrillar assembly, collagen achieves structural stability from the molecular to the tissue level. Collagen biosynthesis is not simply a matter of increasing protein production, but comprises a series of coordinated quality-control steps. Newly synthesized collagen undergoes prolyl and lysyl hydroxylation, glycosylation, propeptide cleavage and covalent cross-linking, which collectively govern its secretion, fibril assembly, receptor recognition and susceptibility to proteolysis. Notably, aging and disease may alter collagen-subtype composition and cross-linking patterns without substantially disrupting the core biosynthetic machinery of the endoplasmic reticulum. Consequently, extracellular matrices with similar collagen abundance can exhibit markedly different mechanical properties and biological activities owing to differences in collagen maturation and molecular organization [23,24,25].

Collagen provides the principal structural framework of the extracellular matrix, thereby determining tissue tensile strength and preserving tissue architecture. Beyond this mechanical role, it regulates a broad range of cellular behaviors through interactions with collagen receptors and matrix-associated proteins [26]. Under physiological conditions, collagen participates in wound healing and tissue repair. Multiple studies have shown that collagen can facilitate tissue regeneration [27,28] by mediating cell adhesion, promoting cell growth and differentiation and modulating the local immune microenvironment [29]. These properties have already supported translational and clinical applications. By contrast, dysregulated collagen synthesis, cross-linking and degradation are closely associated with tumor-cell adhesion and invasion [30,31], as well as tissue fibrosis and matrix stiffening [32,33]. Inflammatory signals can increase MMP expression and alter TIMP-mediated inhibition, thereby changing the extent and timing of collagen proteolysis [34]. Collagen cleavage alters matrix structure and exposes previously hidden sites or releases fragments that influence inflammatory-cell recruitment and tissue repair [35,36]. Once recruited, inflammatory cells release proteases and cytokines that promote further matrix breakdown and sustain local inflammation [36].

In the cardiovascular system, the consequences of these structural and signaling changes depend on the anatomical site, disease stage and timing of repair. Following acute myocardial infarction, orderly collagen deposition contributes to scar formation and helps prevent ventricular rupture [37,38]. However, persistent excessive deposition, aberrant cross-linking, and maladaptive collagen remodeling promote myocardial fibrosis [39], which contributes to left ventricular dilatation and chronic heart failure [40]. Collagen remodeling also shapes the development and progression of atherosclerosis. Increased collagen content within the vascular wall, a reduced elastin-to-collagen ratio, and enhanced cross-linking mediated by advanced glycation end products (AGEs) and lysyl oxidase (LOX) drive arterial stiffening and the progression of atherosclerosis [41]. Although collagen initially stabilizes the fibrous cap, excessive degradation at later stages increases plaque vulnerability [42,43]. Clinically, collagen-related biomarkers also hold value for disease progression prediction and monitoring. Biomarkers such as procollagen type I C-terminal propeptide (PICP) and procollagen type III N-terminal propeptide (PIIINP) may help reflect the burden of myocardial fibrosis [44]. In addition, MMPs, LOX, and collagen-based scaffolds have emerged as important directions in antifibrotic therapy and regenerative medicine [45]. Nevertheless, the long-term clinical benefits of interventions specifically targeting collagen cross-linking or MMP activity remain insufficiently studied and have yet to be fully established. Recent studies underscore the need to distinguish collagen quantity from collagen quality in cardiovascular disease. Indiscriminate suppression of collagen synthesis may compromise healing of the infarct scar, whereas targeting pathological cross-linking or maladaptive collagen turnover could reduce tissue stiffening while preserving the collagen required for structural integrity [8,46].

## 3. Expression of Collagens in Different Tissues

### 3.1. Tissue-Level Collagen Gene Expression Across Reference Tissues

**Figure 1:**
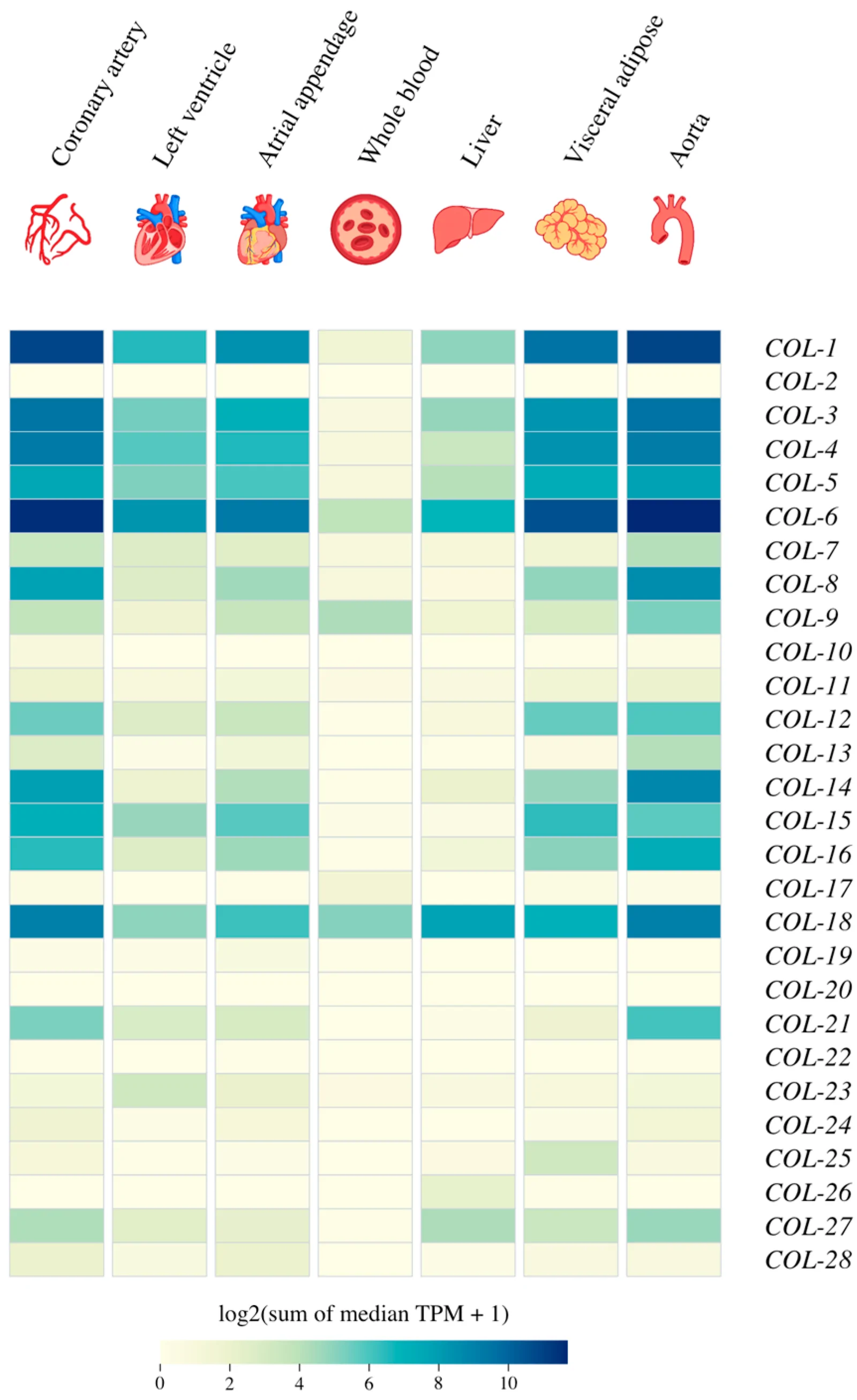
Tissue-specific expression of collagen genes in human cardiovascular tissues. Tissue-specific gene expression data, expressed as transcripts per million (TPM), from the GTEx v11 project are presented as a heat map. The expression profiles of genes encoding the 28 human collagen types are compared across the coronary artery, left ventricle, left atrial appendage, whole blood, liver, visceral adipose tissue (omentum) and aorta. For each collagen type (COL1–COL28), official tissue-level median transcripts per million (TPM) values were summed across the corresponding α-chain genes. Colors and color-bar values represent log2(sum of median TPM + 1), with darker blue indicating higher expression.

Public transcriptomic resources provide a valuable framework for defining the baseline expression landscape of collagen family members across selected cardiovascular and non-cardiovascular tissues. GTEx v11 and the Heart Cell Atlas v2 were selected to provide complementary tissue-level and cardiac cell-type expression profiles. GTEx enables comparisons across arterial, cardiac and reference tissues, whereas the Heart Cell Atlas identifies potential cellular sources of collagen transcripts in the left ventricle. We therefore examined tissue transcriptomic data from the Genotype-Tissue Expression project, release v11 (GTEx v11) [13], available at https://www.gtexportal.org/. The resulting heat map of COL-1–COL-28 revealed marked differences among the coronary artery, left ventricle, atrial appendage, whole blood, liver, visceral adipose tissue (omentum) and aorta (Figure 1). Among the seven tissues examined, the coronary artery and aorta showed comparatively high aggregate transcript levels for COL-1, COL-3, COL-4, COL-6, COL-8, COL-14 and COL-18. This arterial expression pattern may reflect the vessel wall’s requirements for mechanical support and cell–matrix interactions [47]. Coordinated deformation of the coronary artery wall depends on both the interstitial fibrillar scaffold formed by types I and III collagen and the contributions of types IV and XVIII collagen to maintaining pericellular basement membranes and their connections to the interstitial matrix [48]. Type XIV collagen regulates collagen fibril assembly and organization, and studies in mouse myocardia indicate that this process is important for maintaining the structural and functional integrity of the collagen network [49]. These components help maintain arterial strength and compliance under hemodynamic and mechanical stress. In disease, alterations in this matrix may also influence vascular remodeling and fibrous-cap stability.

The left ventricle and atrial appendage also expressed multiple collagen family members, although their overall expression was lower than that in the coronary artery. COL-1, COL-3, COL-4, COL-5, COL-6 and COL-18 were among the more prominent members in cardiac tissues. Unlike the layered arterial wall, myocardial collagen forms a matrix network that surrounds and connects cardiomyocytes, groups of myocytes and microvessels. Three-dimensional imaging of normal rat ventricles shows that perimysial collagen extends along myocardial layers and connects adjacent layers [50]. This arrangement provides structural support and helps coordinate tissue deformation during contraction and relaxation. Higher expression of some collagen types in the atrial appendage than in the left ventricle may reflect differences in cellular composition and matrix maintenance. The adult human heart atlas identifies differences in both cell proportions and the collagen expression profiles of fibroblast subpopulations between atria and ventricles [51]. Spatial multiomics further reveals distinct cellular communities and local microenvironments across cardiac regions. Thus, expression differences between the atrial appendage and left ventricle may reflect regional matrix organization and do not necessarily indicate differences in fibrosis. By contrast, genes encoding most collagen types showed low expression in whole blood, consistent with its lack of a fixed interstitial scaffold.

Visceral adipose tissue also showed substantial expression of several collagen types, particularly I, III, IV and VI. The liver displayed a distinct profile, with type XVIII showing the highest aggregate transcript level among the collagen types examined in this tissue. Although most collagen types showed low expression in whole blood, types IX and XVIII retained relatively prominent signals. These patterns extend the comparison beyond arterial and myocardial tissues and highlight tissue-dependent differences in collagen transcript expression.

Together, these data demonstrate tissue-dependent variation in collagen transcript expression across the seven tissues examined. However, bulk GTEx profiles combine signals from heterogeneous cell populations and do not directly measure collagen protein abundance or matrix deposition. To investigate the cellular contributors to cardiac expression patterns, we next examined single-cell and single-nucleus transcriptomic data from the Heart Cell Atlas v2.

### 3.2. Cell-Type Expression Within the Left Ventricle

**Figure 2:**
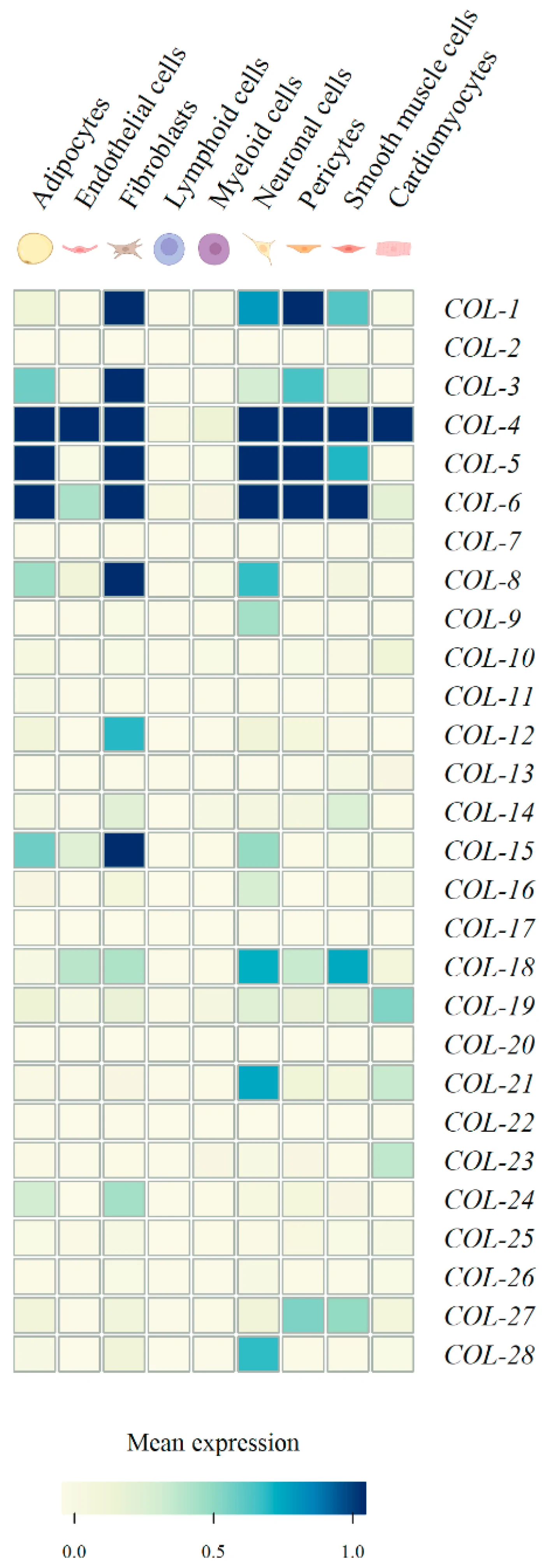
Cell type-specific expression patterns of collagen genes in the human left ventricle. Gene expression of all 28 human collagen types across different left-ventricular cell populations from the single-cell RNA-sequencing dataset of the Heart Cell Atlas is plotted as a heat map [14]. The cell types analyzed include adipocytes, endothelial cells, fibroblasts, lymphoid cells, myeloid cells, neuronal cells, pericytes, smooth-muscle cells and cardiomyocytes. Color intensity indicates relative expression, with darker blue representing higher expression.

To resolve the cellular basis of these tissue-level differences, single-cell and single-nucleus transcriptomic data from the Heart Cell Atlas v2 [14] (https://www.heartcellatlas.org/) were examined across major cardiac cell types (Figure 2). Fibroblasts displayed the broadest and generally strongest collagen expression profile, with prominent signals for COL-1, COL-3, COL-4, COL-5, COL-6, COL-15 and COL-18. This pattern supports fibroblasts as the principal transcriptional contributors to cardiac extracellular matrix production and remodeling. Notably, COL-4 showed relatively high expression across several cell types, including endothelial cells, pericytes, smooth-muscle cells and cardiomyocytes. Its broad distribution is consistent with type IV collagen serving as a core basement-membrane component at cardiac microvascular and cell–matrix interfaces. Adipocytes, neuronal cells, pericytes and smooth-muscle cells also exhibited distinct collagen repertoires, suggesting coordinated matrix maintenance by vascular support and other interstitial cells. By contrast, most collagen transcripts were expressed at low levels in lymphoid and myeloid cells. Cardiomyocytes likewise showed limited overall expression, although selected members retained comparatively distinct signals.

The Heart Cell Atlas v2 complements the tissue-level observations from GTEx by resolving their underlying cellular architecture. The cardiac collagen network appears to be centered on fibroblasts, with coordinated contributions from vascular-wall and other interstitial cells. However, the heat map represents relative transcript abundance and does not directly measure collagen synthesis, secretion or extracellular deposition. These interpretations therefore require further validation using spatial transcriptomic and protein-level evidence.

## 4. The Role of Collagen in Human Cardiovascular Diseases

### 4.1. Collagen in Atherosclerosis and Coronary Artery Disease

**Figure 3:**
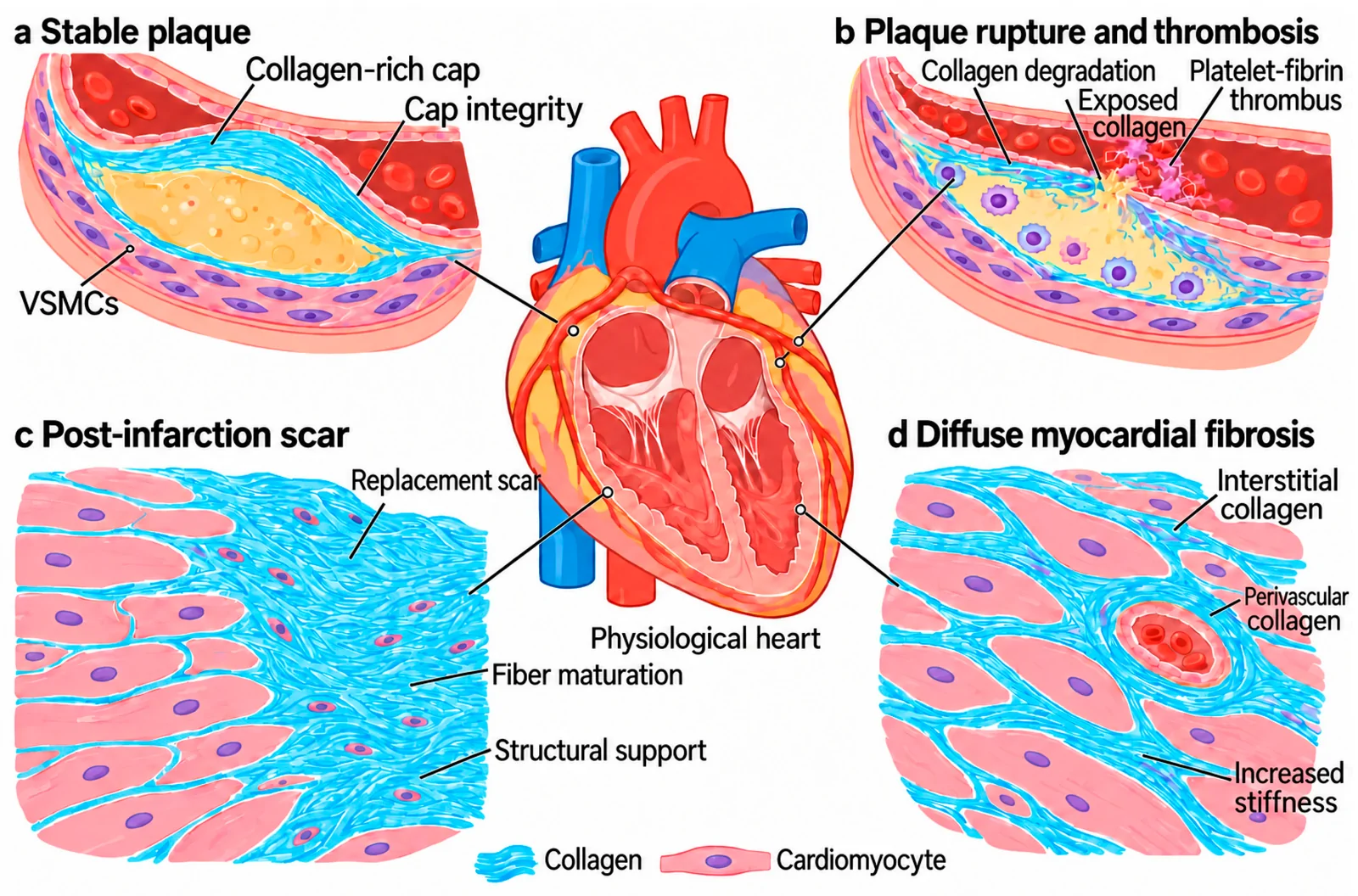
Context-dependent roles of collagen in coronary plaque stability, thrombosis and myocardial remodeling. (**a**) In stable atherosclerotic plaques, an organized collagen-rich fibrous cap, supported by VSMCs. (**b**) Collagen degradation weakens the fibrous cap and increases susceptibility to rupture. (**c**) After myocardial infarction, collagen deposition replaces lost myocardium with scar tissue. Collagen-fiber organization and maturation provide structural support and help maintain ventricular-wall integrity. (**d**) Excessive interstitial and perivascular collagen accumulation contributes to diffuse myocardial fibrosis and increased tissue stiffness.

Changes in collagen expression in coronary artery disease (CAD) are primarily involved in three interconnected pathological processes: atherosclerotic plaque remodeling, the development of acute coronary events, and post-myocardial infarction (MI) repair and fibrosis. CAD is not merely a consequence of lipid accumulation, but rather a complex disorder characterized by inflammation, endothelial dysfunction, vascular smooth-muscle cell (VSMC) migration and proliferation, fibroblast activation, and extracellular matrix (ECM) remodeling [52]. As a major structural component of the ECM, collagen exerts context-dependent effects during this process. On the one hand, it helps maintain tissue architecture and mechanical stability; on the other hand, excessive deposition or abnormal cross-linking may contribute to vascular stiffening, myocardial fibrosis, and adverse ventricular remodeling [53]. The context-dependent roles of collagen in plaque stability, plaque rupture-associated thrombosis, post-infarction scar formation and myocardial fibrosis are illustrated in Figure 3.

Collagen is a key ECM component of the fibrous cap of atherosclerotic plaques. Its abundance, spatial distribution, fiber organization, degradation, and cross-linking collectively determine plaque stability. As atherosclerosis progresses, the ECM composition of the arterial wall undergoes substantial remodeling, with collagen gradually becoming one of the dominant structural proteins within the plaque. Previous studies have shown that type I and type III collagens are abundant in atherosclerotic lesions. Type III collagen accounts for a substantial proportion of the collagen in carotid, femoral and aortic plaques and is predominantly present in a fully processed, cross-linked mature form, indicating its incorporation into a relatively stable collagen matrix within the plaque [54]. Early organ-culture studies also demonstrated that the proportion of newly synthesized type III collagen is higher in atherosclerotic tissue than in normal aorta, indicating a shift in collagen subtype expression toward a reparative and fibrotic matrix phenotype during lesion progression [55]. Histologically, stable plaques are generally characterized by a thick fibrous cap, abundant collagen, and relatively high VSMC content, whereas vulnerable plaques typically exhibit thin caps, reduced collagen content, increased inflammatory cell infiltration, and an enlarged necrotic core [56,57].

VSMCs are a major cellular source of collagen in atherosclerotic plaques and exhibit substantial phenotypic plasticity. In addition to adopting a contractile phenotype that maintains vascular tone, VSMCs can transition to a synthetic phenotype characterized by proliferation, migration and matrix synthesis, or acquire inflammatory and osteogenic features [58]. VSMCs that migrate into the intima can transform into a synthetic phenotype, secreting type I and III collagen and participating in the formation of fibrous caps. However, chronic inflammation, excessive lipid exposure, and cellular senescence can promote VSMC apoptosis or phenotypic transition, leading to impaired matrix synthesis, reduced collagen production, and weakening of the fibrous cap [56]. For example, trimethylamine N-oxide (TMAO) has been shown to reduce fibrous cap thickness and collagen deposition by downregulating genes involved in ECM organization and collagen formation in VSMCs, thereby increasing plaque vulnerability [59]. In turn, collagen and the surrounding extracellular matrix can influence VSMC phenotype. VSMCs cultured on type IV collagen exhibit higher myocardin expression, increased SRF binding to CArG elements in contractile protein gene promoters, and enhanced transcription of contractile genes, suggesting that type IV collagen supports the maintenance of the VSMC contractile gene expression program [60]. Studies using human aortic tissue have shown that SOX9 reduces the abundance of basement membrane-associated components, including type IV and VI collagens, while promoting extracellular vesicle-mediated deposition of the collagen-modifying enzyme LH3 into the matrix, thereby increasing matrix stiffness. The resulting matrix suppresses VSMC proliferation and increases DNA damage, whereas matrix produced following SOX9 depletion allows senescent cells to re-enter the cell cycle [61]. Inflammation within plaques further destabilizes the collagen-rich fibrous cap by accelerating collagen degradation. ADAMTS-7 is upregulated in the fibrous caps of unstable plaques and enhances the degradation of TIMP-1, thereby weakening its inhibitory effect on MMP-9 [42]. This process enhances MMP-9-mediated matrix degradation, potentially contributing to plaque destabilization. MMP-9 primarily degrades gelatin and basement-membrane components, including type IV collagen [62], whereas the initial cleavage of native fibrillar collagens, particularly types I and III, is mediated mainly by interstitial collagenases such as MMP-1, MMP-8 and MMP-13 [63]. The balance between fibrillar collagen synthesis and proteolytic degradation is therefore a key determinant of fibrous cap integrity and plaque stability.

**Figure 4:**
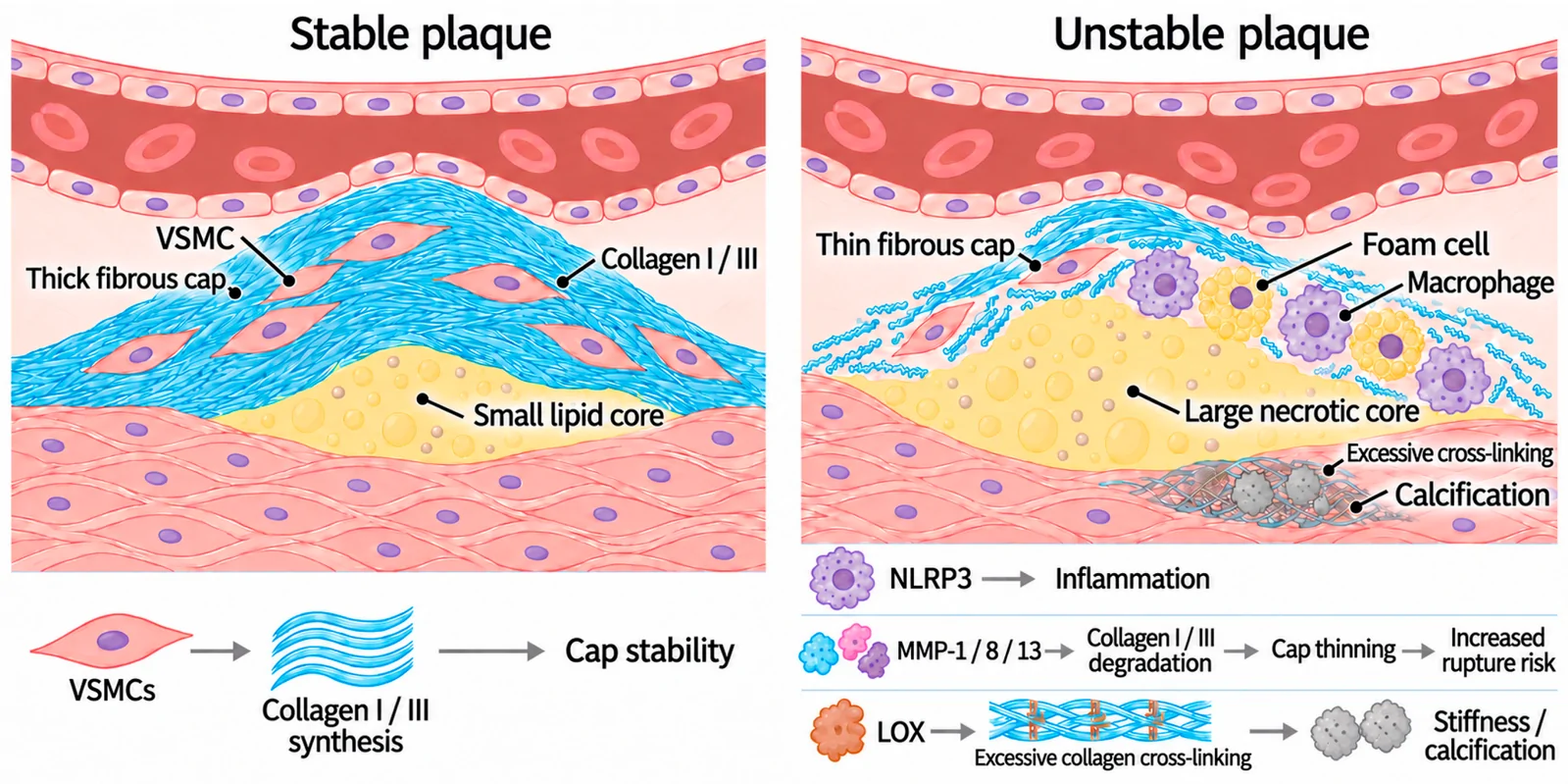
Collagen remodeling in atherosclerotic plaque stability. VSMC-derived types I and III collagen support fibrous-cap integrity, whereas inflammation and MMP-mediated collagen degradation promote cap thinning and plaque vulnerability. Excessive LOX-mediated collagen cross-linking can increase matrix stiffness and contribute to vascular calcification. VSMCs, vascular smooth-muscle cells; MMPs, matrix metalloproteinases; LOX, lysyl oxidase.

In vivo studies further support a close association between increased plaque collagen content, improved collagen-fiber organization and greater plaque stability. Colchicine, for example, suppresses foam-cell formation and NLRP3 inflammasome activation, thereby reducing lipid accumulation and necrotic-core formation in unstable plaques. It also increases plaque collagen content and the ratio of fibrous-cap thickness to necrotic-core area, collectively favoring plaque stabilization [64]. A growing body of experimental evidence similarly indicates that pharmacological and bioactive compounds can reprogram macrophage phenotypes, restrain pro-inflammatory responses and promote reparative states. These changes enhance collagen synthesis and extracellular-matrix remodeling, facilitating fibrous-cap formation and plaque stabilization [65,66]. Increased collagen synthesis, however, does not invariably confer benefit. Some compounds stimulate collagen production while simultaneously increasing macrophage infiltration and plaque burden, suggesting that they may accelerate lesion progression despite collagen accumulation [67]. Reducing inflammation can also increase plaque collagen content. Guizhi Tongluo tablets, salvianolic acid B and MCL increase collagen deposition by inhibiting Piezo1–NLRP3-mediated pyroptosis, NF-κB–NLRP3 signaling and macrophage ferroptosis, respectively [68,69,70]. In human atherosclerotic plaques, proprotein convertase subtilisin/kexin type 9 (PCSK9) inhibitors reduce the abundance of inflammation-associated proteins and increase collagen content, suggesting that their anti-inflammatory effects and modulation of extracellular-matrix remodeling may jointly contribute to plaque stabilization [71]. Nevertheless, collagen deposition is not uniformly protective. Excessive collagen cross-linking mediated by lysyl oxidase (LOX) increases matrix stiffness and can promote vascular calcification and plaque progression [72,73]. Collagen remodeling in atherosclerotic plaques is therefore both stage-dependent and context-dependent. During early lesion development or plaque stabilization, controlled collagen synthesis and deposition reinforce the fibrous cap. By contrast, in advanced lesions with persistent inflammation, accelerated collagen degradation, impaired matrix synthesis by vascular smooth-muscle cells and aberrant collagen cross-linking can each drive the transition towards a vulnerable plaque phenotype. The balance between collagen synthesis, proteolytic degradation and cross-linking, and its implications for fibrous-cap integrity and vascular remodeling, are summarized in (Figure 4).

### 4.2. Myocardial Infarction and Heart Failure

Once the fibrous cap ruptures or endothelial integrity is disrupted, subendothelial collagen becomes exposed to circulating blood. Under these conditions, collagen rapidly shifts from a protective structural component to a potent thrombogenic stimulus. Human atherosclerotic plaques contain abundant type I and type III collagens, both of which can be recognized by the platelet collagen receptor glycoprotein VI (GPVI). In vitro studies have shown that blockade of GPVI or selective masking of type I and type III collagens within plaques markedly reduces plaque-induced platelet adhesion and aggregation [74,75]. Subsequent studies have demonstrated that coronary thrombosis after plaque rupture is driven by the coordinated actions of collagen-mediated platelet activation and tissue factor-mediated coagulation. When human atherosclerotic plaque material is exposed to flowing blood, plaque collagen induces rapid platelet adhesion and aggregation through GPVI within the first minute. Thereafter, plaque-associated tissue factor promotes thrombin generation and fibrin formation, converting the initial platelet aggregate into a stable, potentially occlusive thrombus [76]. This two-stage mechanism helps explain why plaques characterized by intense inflammation, fibrous cap thinning, and disruption of the collagen barrier are particularly likely to precipitate acute coronary syndromes following rupture. Clinical observations are consistent with this mechanism. Compared with patients with stable angina, those presenting with acute coronary syndromes exhibit higher levels of platelet surface GPVI. Moreover, increased GPVI expression correlates with markers of platelet activation and has been independently associated with the risk of acute coronary events [77,78].

Therefore, collagen has a dual pathophysiological role in CAD. In intact plaques, an adequate amount of well-organized collagen reinforces the fibrous cap and reduces the likelihood of rupture. Following plaque disruption or endothelial injury, however, exposed collagen, particularly type I and type III collagens, initiates arterial thrombosis through GPVI-dependent platelet activation and cooperates with tissue factor-driven coagulation to promote acute coronary events such as MI. Collagen is therefore not only an ECM marker of plaque stability, but also a critical effector linking plaque rupture to platelet activation and coronary thrombosis.

After acute MI, collagen expression within the myocardial interstitium exhibits marked temporal, spatial, and subtype-specific changes. During the early phase of infarction, myocardial necrosis initiates a robust inflammatory response, characterized by activation of inflammasome signaling and increased IL-1β activity. At the same time, the interstitial collagen fraction begins to rise, suggesting that inflammation participates in initiating subsequent ECM remodeling and collagen deposition [79]. Transcriptomic studies have further demonstrated rapid alterations in remodeling of ECM-related genes after acute MI, including increased expression of the classical fibrillar collagen genes COL1A1 and COL3A1, which provide the molecular basis for subsequent scar formation [80,81]. As repair progresses, collagen accumulation becomes increasingly prominent within the infarcted myocardium. In reperfused MI models, deposition of type I and type III collagens is markedly increased in necrotic myocardial regions, whereas changes in salvaged regions are comparatively modest. These findings indicate that post-infarction collagen remodeling is not only time-dependent but also spatially heterogeneous [82]. Traditionally, the post-MI scar has been considered to consist predominantly of type I and type III collagens. More recent evidence, however, indicates that several non-classical collagen subtypes also contribute to scar development and structural maturation. Although type V collagen is relatively limited in abundance, it participates in the regulation of scar size and structural stability [83]. Moreover, macrophage-derived type V collagen may influence early collagen assembly and alignment after MI [11]. Other fibrillar and non-fibrillar collagens, including types II, VIII, XI, and XII, show increased gene expression during post-infarction scar formation, and their corresponding proteins have been detected in chronic human myocardial infarction scars [84]. Type XV collagen appears to exert a protective role in maintaining post-MI cardiac structure, scar resistance, and left ventricular function. Its deficiency results in upregulation of fibrosis-associated genes in the remote myocardium, increased tissue stiffness, and abnormalities in scar ultrastructure [85]. At the protein level, post-MI remodeling is accompanied by dynamic changes in ECM protein composition, collagen content and matrix mechanical properties. A study combining hydroxyproline assays, second harmonic generation (SHG) imaging and two-photon excited autofluorescence assessed collagen content, fiber organization and cross-linking status in the extracellular matrix, suggesting that ECM mechanical properties depend not only on collagen abundance but also on its organization and maturation state [86]. Further evidence indicates that collagen dysfunction is not determined solely by changes in total collagen abundance. Studies of cellular communication network factor 1 (CCN1) have shown that even when type I collagen (COL1A1) synthesis and total scar collagen remain largely unchanged, reduced levels of the collagen-processing and stabilizing enzymes P4HA1, PLOD1 and PLOD2, together with abnormalities in fiber alignment, fibril diameter and packing, can compromise scar integrity and increase the risk of cardiac rupture [37]. Thus, the maturation and spatial organization of collagen fibers are critical determinants of repair quality after myocardial infarction. Several molecular pathways contribute to post-infarction fibrotic remodeling by regulating collagen synthesis, deposition and degradation. NAT10 enhances N4-acetylcytidine (ac4C) modification of TGFBR1 mRNA, thereby increasing transforming growth factor β (TGF-β) signaling, upregulating the collagen-related genes Col1a1 and Col3a1, and promoting the deposition of type I and type III collagen during post-infarction fibrosis [87]. By contrast, thymic stromal lymphopoietin (TSLP) modulates immune-cell recruitment and the inflammatory microenvironment, limiting excessive collagen deposition and attenuating maladaptive fibrosis after myocardial infarction [88]. Anthrax toxin receptor 1 (ANTXR1)-dependent collagen turnover and extracellular-matrix remodeling also contribute to heart failure and post-infarction scar formation. Aberrant ANTXR1 activation disrupts collagen homeostasis and promotes adverse scar remodeling, whereas inhibition of ANTXR1 signaling improves collagen organization within the infarcted region and reduces late TGF-β-associated matrix accumulation [89]. Together, these findings identify the dynamic balance among collagen synthesis, post-translational maturation, degradation and spatial organization as a central determinant of stable scar formation and functional cardiac recovery. Therapeutic modulation of collagen metabolism may therefore offer a strategy for limiting myocardial fibrosis, although its efficacy is likely to depend on the timing and specific component of collagen remodeling being targeted.

## 5. Clinical Implications and Genetic Diversity

Available evidence suggests that polymorphisms in collagen-related genes may contribute to CAD and acute coronary events by affecting vascular ECM composition, fibrous cap strength, and susceptibility to plaque rupture. The most extensively investigated locus is the COL4A1–COL4A2 gene cluster on chromosome 13q34. Large-scale genome-wide association studies first identified the single-nucleotide polymorphism rs4773144, located within the third intron of COL4A2, as a susceptibility locus for CAD. The risk allele G was associated with increased CAD risk [90,91]. Functional studies subsequently demonstrated that the rs4773144-G allele reduces transcriptional expression of COL4A1 and COL4A2 in VSMCs and endothelial cells. Compared with individuals carrying the A/A genotype, those with the G/G genotype exhibit the lowest expression of type IV collagen, together with increased VSMC apoptosis, reduced type IV collagen abundance in coronary atherosclerotic plaques, and thinning of the fibrous cap. Among patients with angiographically confirmed CAD, the G/G genotype was also associated with a higher incidence of MI. These findings suggest that rs4773144 may increase the risk of plaque rupture and subsequent thrombotic events by compromising basement-membrane integrity and plaque stability [92]. However, the association is not uniform across all clinical phenotypes. In a case–control study of patients with unstable angina, rs4773144 genotype distribution was not significantly associated with disease risk, suggesting that its effect may be more closely related to plaque rupture leading to MI or may depend on population background, disease definition, and sample size [93].

In addition to type IV collagen, the COL3A1 gene encoding type III collagen is also associated with vascular integrity and coronary events. Type III collagen is the main component of reticular fibers and can be assembled with type I collagen to form atypical collagen fibrils, which participate in regulating the tissue structure of fibers and maintaining the integrity of arterial walls [94]. In atherosclerotic plaques, type III collagen participates in the formation of fibrous caps, and when exposed to circulating blood it can also mediate platelet adhesion and activation [95]. Type III collagen is an important structural component of the arterial wall and plaque fibrous cap, and it also serves as a ligand involved in platelet adhesion and activation. Early studies of a tandem repeat polymorphism within intron 25 of COL3A1 identified allele-specific differences in CAD risk and outcomes after acute coronary syndromes. In cohorts including patients with MI and unstable angina, carriers of the COL3A1-4 allele showed lower risks of acute coronary syndrome and stable angina. In the OPUS-TIMI-16 acute coronary syndrome cohort, this allele was also associated with a reduced risk of composite ischemic events. Conversely, other COL3A1 alleles were associated with increased composite endpoint risk and may have influenced responses to antiplatelet treatment [95]. By contrast, pathogenic COL3A1 variants in vascular Ehlers–Danlos syndrome (vEDS) have better-established functional consequences. Glycine substitutions within the triple-helical domain and splice variants causing in-frame exon skipping can disrupt collagen folding and assembly, whereas variants leading to haploinsufficiency reduce the production of normal type III collagen. These defects compromise arterial-wall integrity and increase the risk of dissection and rupture [94]. Recent studies using patient-derived cells have further shown that specific glycine substitutions cause type III collagen misfolding, intracellular retention and reduced extracellular deposition [96]. Matrix produced by patient-derived cells also exhibits altered composition and viscoelastic properties and can influence endothelial cell migration [97]. Nevertheless, compared with the COL4A1–COL4A2 locus, evidence for COL3A1 polymorphisms is based largely on earlier studies and has not yet been sufficiently replicated in large, contemporary cohorts; therefore, its clinical predictive value remains uncertain. Additional studies have implicated other COL4A1–COL4A2-region variants, COL6A3, COL1A1 and COL15A1 in CAD or related atherosclerotic phenotypes, although the strength and scope of evidence vary across studies [90,92,93,95,98,99,100,101,102]. These findings, together with the variants discussed above and their evidence limitations, are summarized in Table 1.

In summary, genetic studies implicate several collagen genes in CAD and related atherosclerotic phenotypes, with varying levels of association and functional evidence. The biological relevance of these variants extends beyond changes in collagen abundance alone. By altering basement-membrane integrity, VSMC survival, fibrous cap collagen content, and potentially platelet responses to exposed collagen, these genetic variants may influence plaque development, rupture, and the occurrence of acute coronary events. At present, the COL4A1–COL4A2 locus is supported by the most consistent genetic and functional evidence, whereas associations involving other collagen subtypes remain fragmented. Future studies should validate these findings in large, ethnically diverse populations and integrate genetic data with plaque imaging, tissue-level protein expression, and longitudinal clinical outcomes. Thus, the clinical interpretation of collagen-associated genetic variation will require evidence integrated across multiple molecular scales. A given risk allele may affect transcription, post-transcriptional regulation, protein processing, or the way collagen is incorporated into the extracellular matrix, without necessarily producing a proportional change in total collagen abundance. Future studies should combine genetic analyses with single-cell or cell-resolved expression profiling, quantitative matrix imaging and longitudinal clinical outcomes. Only through such integrated approaches can individual collagen variants be robustly incorporated into polygenic risk models or used to guide patient-specific prevention and therapeutic strategies [103,104].

## 6. Conclusions

Previous studies and publicly available omics data implicate collagen remodeling in atherosclerotic plaque stability, coronary thrombosis and post-infarction repair. By integrating tissue- and cell-type expression profiles, genetic associations and experimental evidence, this review connects the molecular regulation of collagen with matrix architecture and disease outcomes, emphasizing the importance of collagen maturation, spatial organization and functional properties alongside changes in abundance. Whether collagen exerts protective or pathological effects depends not only on its abundance but also on its molecular subtype, cellular source and disease stage. Single-cell and single-nucleus transcriptomics help identify the cellular sources of collagen transcripts, but mRNA abundance alone cannot resolve collagen deposition, cross-linking, degradation or tissue mechanics. Future studies should therefore integrate transcriptomics, spatial proteomics, in situ matrix imaging and tissue mechanical measurements to determine how changes in gene expression translate into alterations in matrix structure and function. Genetic association studies provide complementary evidence, as illustrated by the COL4A1–COL4A2 locus, although many associations and their functional mechanisms require independent validation.

Collagen is both a structural component regulated by upstream signals and an active modulator of cell behavior through interactions with cell-surface receptors and the activities of collagen-derived bioactive fragments. However, the contributions of these processes across anatomical sites and disease stages, and their feedback effects on local matrix turnover and tissue mechanics, remain incompletely understood in CAD. Future studies should combine local identification of collagen-derived fragments with receptor functional analyses and tissue mechanical measurements to distinguish their roles as markers of matrix turnover from their contributions as disease mediators. These studies should also establish how the balance among collagen synthesis, maturation and degradation influences fibrous-cap integrity, scar stability and myocardial stiffness. Linking these changes to longitudinal clinical outcomes could help determine when to preserve collagen deposition and maturation, limit excessive cross-linking or inhibit platelet activation by exposed collagen, thereby informing the development of collagen-related biomarkers and the selection of therapeutic targets.

## Figures and Tables

**Table 1:** Collagen gene variants associated with coronary artery disease and related atherosclerotic phenotypes.

Gene	Variant	Study Population and Disease Phenotype	Main Findings	Functional Mechanisms and Evidence Limitations	References
*COL4A1*/*COL4A2*	rs4773144	CAD GWAS, vascular-cell studies and coronary-plaque analyses; an unstable-angina study included 232 patients and 144 controls.	The G allele was associated with reduced COL4A1/COL4A2 expression. Plaques from individuals with the G/G genotype had lower type IV collagen content and thinner fibrous caps.	Supported by regulatory and cellular functional evidence; effects may depend on the phenotype and population.	[90,91,92]
*COL4A2*	rs9515203	Imaging GWAS: 24,811 participants in the SIS analysis and 26,000 in the CACS analysis.	The variant was associated with coronary plaque burden and coronary artery calcification in an imaging GWAS.	Noncoding association marker; the underlying causal mechanism remains unclear.	[97]
*COL4A1* region	rs2000660	Imaging GWAS of coronary CT-derived plaque burden involving 24,811 participants.	The variant was associated with coronary plaque burden in an imaging GWAS.	Regional association; genomic proximity alone does not establish a unique effector gene.	[97]
*COL4A1* region	rs11617955	Imaging GWAS of coronary artery calcification involving 26,000 participants.	The variant was associated with coronary artery calcification in an imaging GWAS.	The finding concerns calcification and does not directly quantify the risk of clinical CAD events.	[97]
*COL6A3*	rs11677932	Integration of CAD GWAS, protein and expression QTLs, instrumental-variable analyses, MR and colocalization analyses.	Studies prioritized COL6A3-derived endotrophin as a potential contributor to CAD, including obesity-related CAD.	Supports a regulatory pathway and a potential therapeutic target; the variant is not an established cause of monogenic CAD, and therapeutic efficacy remains unproven.	[98,99]
*COL3A1*	Intronic tandem-repeat alleles, including COL3A1-4	703 patients with ACS and an independent OPUS cohort of 924 patients; additional participants included patients with stable angina and controls.	Early studies suggested that COL3A1-4 carriers had lower odds of ACS and fewer composite events during follow-up.	Early candidate-marker evidence requiring replication in contemporary cohorts and sequence-level allele mapping.	[94]
*COL1A1*	Sp1-binding-site polymorphism; designated −1245 G/T in the original study	136 MI survivors and 212 age-matched controls.	Genotype distributions differed between cases and controls.	Small candidate study with potential survivor-selection bias; a standardized variant identifier was not established in this review.	[100]

Abbreviations: CAD, coronary artery disease; GWAS, genome-wide association study; SIS, segment involvement score; CACS, coronary artery calcium score; CT, computed tomography; QTL, quantitative trait locus; MR, Mendelian randomization; ACS, acute coronary syndrome; MI, myocardial infarction.

## Data Availability

The public datasets used for Figure 1 and Figure 2 are available from the GTEx Portal (https://www.gtexportal.org/) and Heart Cell Atlas v2 (https://www.heartcellatlas.org/), respectively.
